# Communication skills training in advance care planning: a survey among medical students at the University of Antwerp

**DOI:** 10.1186/s12904-022-01042-y

**Published:** 2022-08-31

**Authors:** Mick van de Wiel, Katrien Bombeke, Annelies Janssens

**Affiliations:** 1 Department of Thoracic Oncology, Antwerp University Hospital (UZA), Drie Eikenstraat 655, 2650 Edegem, Belgium; 2 Department of Primary and Interdisciplinary Care, Skills Lab, University of Antwerp, Campus Drie Eiken, Universiteitsplein 1, 2610 Wilrijk, Belgium

**Keywords:** Palliative care, Advance care planning, Medical education, Communication skills, End-of-life conversations

## Abstract

**Background:**

Palliative care (PC) is a strongly emerging discipline worldwide. Despite efforts to integrate this important topic in the medical curriculum in Belgium, still little time is spent on PC and its implementation during theoretical and practical training.

**Materials & methods:**

We had two cohorts of second master’s year MD students at the University of Antwerp complete a survey compromising a custom-built PC knowledge test and a self-confidence assessment of communicative skills used in end-of-life conversations. We evaluated students’ self-confidence regarding end-of-life-conversations before and after a PC training program. We also explored whether the PC classes enabled the students to adequately reflect on factors that might influence end-of-life conversations with an open-end question about the potential implications of the COVID-19 pandemic on advance care planning (ACP) conversations. Finally, we compared the results of the respondents having enjoyed face-to-face training (cohort 1) with those having received online training only (cohort 2, COVID-19 pandemic).

**Results:**

Although the respondents in both cohorts indicated that the overall curriculum did not pay enough attention to PC training, their average scores on the theoretical questions were good. Feeling confident about their communicative skills in general, they indicated to be less confident when it came to communications concerning PC and ACP in particular. The COVID-19 pandemic was initially equally deemed to impede and facilitate ACP and end-of-life conversations, but after the ACP training class more respondents saw the pandemic as an opportunity to broach end-of-life issues. Finally, we found no differences in scores between online and regular classroom teaching.

**Conclusion:**

Students experience a lack of confidence in communication skills used in end-of-life conversations and ACP. To help improve skills and competencies in conducting end-of-life conversations, it is recommended to have medical students assess PC/ACP training programs regularly and to modify the curriculum and course content based on these outcomes and current developments.

**Supplementary Information:**

The online version contains supplementary material available at 10.1186/s12904-022-01042-y.

## Introduction/background

The aim of palliative care (PC) is to support and manage patients who can no longer benefit from curative therapy, for which approach there is a growing need. It is a broad concept concerning physical, social, psychological, and existential problems, and can, among other components, comprise psychological counseling, social-work interventions, pain treatment, palliative sedation and euthanasia, preferably predefined using advance care planning (ACP). In general, ACP enables individuals at all stages of life, to reflect on their future health care. It is a process in which the patient, their relatives/caregivers and care providers discuss and jointly formulate care goals. These goals are based on what the patient and attending health professionals view as good quality care, is based on the values, norms and preferences of the patient and can always be adapted to fit the patient’s changing situation. They generally include end-of-life wishes and directions to guide end-of-life decisions when the patient is no longer able to express his/her wishes him/herself [1–3]. ACP has been found to reduce unwanted hospitalization, improve the quality of patient-clinician communications, foster patient-specific PC, and increase patient satisfaction and quality of life [4, 5].

The organization and implementation of PC and PC training varies widely across countries [6–11]. Not only theoretical knowledge about PC, but also the acquisition of the proper communication skills is of great importance, particularly to starting physicians [12]. The European Association of Palliative Care (EAPC) is actively striving to include PC early on in the medical curriculum [13, 14]. Despite the fact that many countries, including Belgium, have already integrated the PC component into the undergraduate training program, the amount of time spent on PC education is very limited [6, 8, 10, 15–17].

With the present study, we sought to determine whether our current PC/ACP training program is able to increase students’ self-reported confidence regarding their communication skills. We further assessed whether going online due to the COVID-19 pandemic had affected the outcomes. Lastly, to find out whether students had learned to utilize different conditions to broach the subject of ACP, we gauged their opinions on whether and how the COVID-19 pandemic might influence such conversations. To our best knowledge, there is no evidence concerning the impact of such trainings on self-reported confidence in communication skills.

## Materials & methods

We opted for an exploratory mixed methods design, collecting both quantitative and qualitative data using a purpose-built survey. The study (registration number B300201942197) was approved by the Antwerp University Hospital’s ethics committee. In normal conditions, we have eight teachers facilitating small group PC/ACP communication skills sessions. In 2020, however, the COVID-19 pandemic challenged education systems worldwide and at the University of Antwerp all courses were compulsorily converted to online classes. We used breakout rooms to allow the students to interact with each other and practice their PC/ACP communication skills that were now monitored by two teachers each servicing over 50 undergraduates. During the second master’s year in Belgium, students do not yet have clinical activities. This is the last year of theoretical training after which consecutive clinical clerkships begin. We recruited two cohorts of second master’s year MD students in two consecutive years (2019 and 2020) and asked them to complete a survey before and after the ACP communication skills session.

The survey consisted of four parts (see Additional file 1: Appendix 1). The first part asks for the respondent’s demographic data, while also comprising three questions on the perceived adequacy of PC training. The second section is a test assessing the respondent’s current knowledge about PC using five theoretical questions based on the course content (max. Total score: 11) [18]. The third part of the survey comprised the Dutch translation of the clinical communication skills domain of the Palliative Education Assessment Tool (PEAT), which had them indicate whether they had confidence in being able to discuss relevant topics relating to PC/ACP and end-of-life issues. The fourth part was only offered to the 2nd cohort, given the unfolding COVID-19 pandemic. This part was a qualitative inquiry. We asked the students to explain whether they considered the COVID-19 pandemic an impeding or facilitating factor for engaging in an ACP or end-of-life conversation.

After having provided their informed consent, the students completed the survey shortly prior to taking the ACP communication skills class (T1) and again after approximately 3 weeks, coinciding with the deadline for the PC/ACP paper assignment (T2). The first, 2019 cohort first filled out the paper survey during an on-campus plenary class. For the second assessment they completed an online version from their homes. The respondents in the second, 2020 cohort completed both assessments online with a similar interval. We used “LimeSurvey” to collect the online data, which was then exported to SPSS, version 27 and used for our descriptive analysis.

Independently from each, the first and second authors (MvdW and KB) thematically analyzed the students’ descriptive responses using Excel. In a first phase, they labeled the students’ responses individually using open codes, closely reflecting the terms used by the students, adding notes and interpretations when relevant. In a next step, they compared and categorized the open codes and annotations in overarching categories. Finally, remaining differences in interpretations were discussed in a joint session with three independent researchers (MvdW, KB and AJ) to reach consensus and compile a definitive overview of the different categories.

## Results

**Fig. 1:**
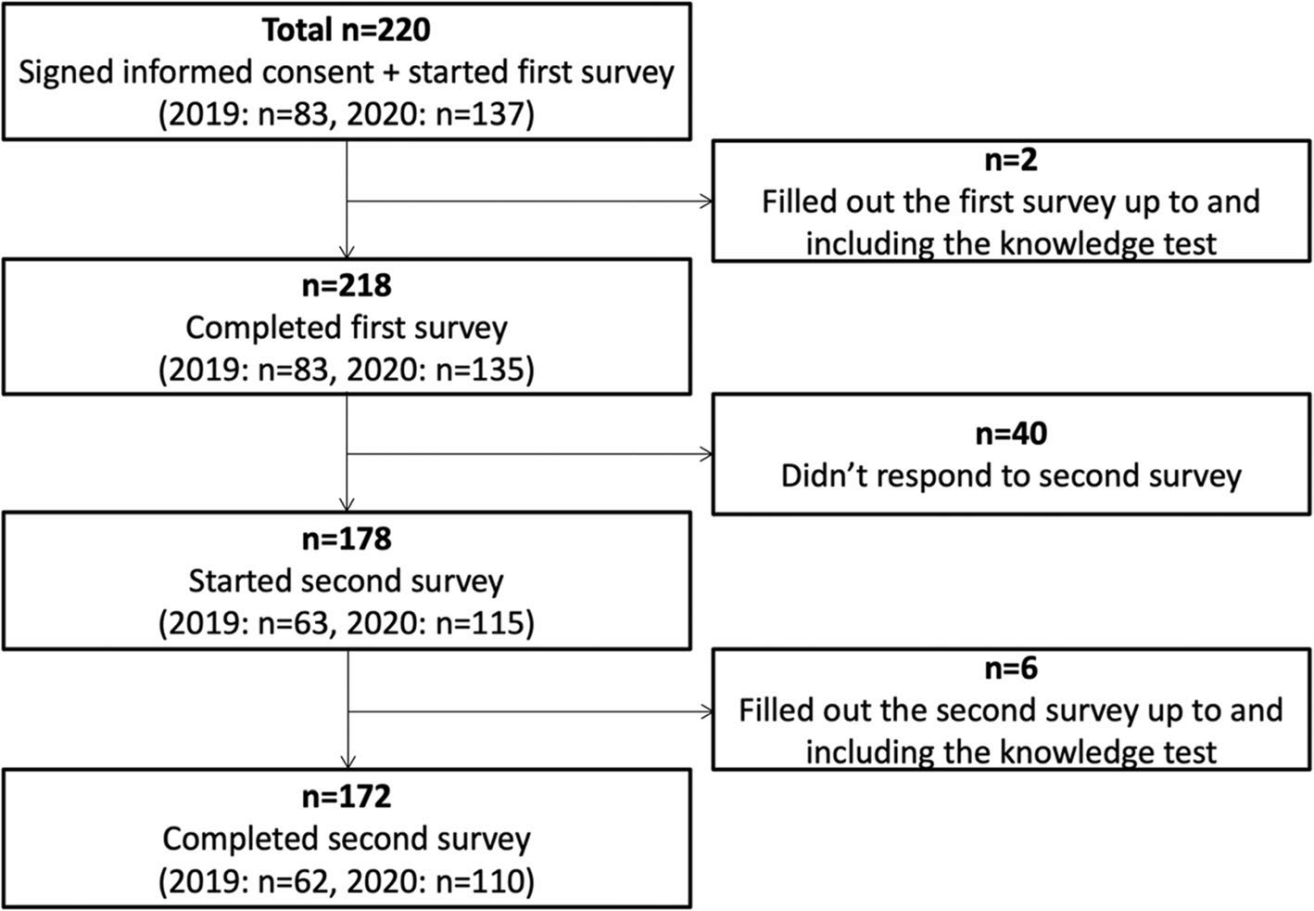
Flow diagram

A total of 231 students were invited and 220 students participated in our study. Table 1 shows their demographics and Fig. 1 a flow diagram detailing the number of respondents at all stages of the study.

**Table 1:** Demographics of the survey respondents

Self-identified gender	C1 (***n*** = 83)	C2 (***n*** = 137)	Total (***n*** = 220)
Male	**34 (41%)**	**53 (39%)**	87 (40%)
Female	**49 (59%)**	**84 (61%)**	133 (60%)
**Age**			
Min-Max (years)	**20–30**	**20–47**	20–47
Mean (years)	**22**	**23**	23
**Religion**			
None	**48 (58%)**	**79 (58%)**	127 (58%)
Catholic	**31 (37%)**	**48 (35%)**	79 (36%)
Islam	**3 (4%)**	**5 (4%)**	8 (3%)
Judaism	**1 (1%)**	**3 (2%)**	4 (2%)
Hinduism	**0**	**1 (< 1%)**	1 (< 1%)
Other	**0**	**1 (< 1%)**	1 (< 1%)
**Preferred Specialty**			
Family doctor	**26 (31%)**	**38 (28%)**	64 (29%)
Specialist/consultant	**48 (58%)**	**99 (72%)**	147 (67%)
No preference as yet	**9 (11%)**	**0**	9 (4%)

*C1* Cohort from 2019 (face-to-face training), C2 Cohort from 2020 (online training)

Table 2 presents the percentages of students having answered the following questions affirmatively: “Do you think that enough time is spent on end-of-life education?”; “Do you feel you have gained enough knowledge from the end-of-life classes?”; “Do you feel that you are able to independently choose the right moment and have a conversation with a patient about advance care planning?”. Prior to the PC classes, the majority (58%) of the respondents in the 2019 cohort found that PC/end-of-life training time suffices; in the 2020 cohort, this was only true for 47%. Having completed the course, three quarters of all respondents indicated being satisfied with the time being dedicated to PC. In both cohorts most students (82 and 77% respectively) indicated that their knowledge regarding PC was insufficient, but after the course, this had increased, with 67 and 70% now stating to have acquired sufficient insight into PC/ACP. Initially, the minority (10 and 12% respectively) deemed they were sufficiently capable of independently broaching ACP and negotiate a plan, but this had increased to 48 and 40% after the PC classes.

**Table 2:** Proportions of respondents having answered “Yes” to the questions gauging the perceived adequacy of the PC training program

	2019 cohort (face-to-face-training)		2020 cohort (online training)	
	T1 (***n*** = 83)	T2 (***n*** = 63)	T1 (***n*** = 137)	T2 (***n*** = 115)
Enough time spent on PC	58%	76%	47%	73%
Sufficient knowledge about PC	18%	67%	23%	70%
Independent clinical experience with ACP	10%	48%	12%	40%

*PC* Palzliative care, *ACP* Advance care planning, T1 = survey prior to the ACP training class, T2 = survey following the ACP class and paper assignment

Table 3 lists the average scores for the PC knowledge test. In general, the students scored well on the test at both timepoints, with the post-course mean scores showing the most improvement relative to the baseline scores in the 2019 cohort.

**Table 3:** PC knowledge test scores for the two cohorts (max. Total score: 11)

	2019 cohort (face-to-face training)		2020 cohort (online training)	
	T1 (***n*** = 83)	T2 (***n*** = 63)	T1 (***n*** = 137)	T2 (***n*** = 115)
Mean	7.4	9.4	8.2	8.6

T1 = survey prior to the ACP class, T2 = survey following the ACP class and paper assignment

As to the students’ self-reported confidence in PC/ACP-related communication skills (see Table 4; domains derived from the PEAT), we found that both in 2019 and 2020 a vast majority of the respondents reported feeling confident about maintaining an empathic attitude towards patients and/or family members and during consultations and respecting other cultures and beliefs both before and after the course. Pre-course confidence on the topics of discussing the patient’s impending death, reporting the patient’s death, coping with a palliative patient and discussing ACP was particularly low (less than 30%) in the 2019 cohort, while in the 2020 cohort coping with a palliative patient, discussing ACP and, withholding life-prolonging treatment appeared to pose the most problems. After the course, confidence had improved in most domains, with resolving conflicts or negotiating conflicts being the only topic showing a post-course drop in the 2019 cohort.

**Table 4:** Proportions of respondents reporting confidence in their communication skills for palliative care (PC) and advance care planning (ACP) dimensions

I feel confident that I am able to …	2019 cohort (face-to-face training)		2020 cohort (online training)	
	T1 (***n*** = 83)	T2 (***n*** = 62)	T1 (***n*** = 135)	T2 (***n*** = 110)
… empathize with the patient and/or family members/caregiver(s)	99%	98%	99%	99%
… empathically engage in an interview/consultation	98%	98%	95%	98%
… respect and convey knowledge about different cultures and beliefs	95%	97%	85%	92%
… ask for informed consent	77%	92%	84%	87%
… cooperate and communicate well within a multidisciplinary team	71%	87%	78%	93%
… break bad news/conduct a ‘bad news’ consultation	70%	68%	71%	70%
… derive relevant clinical information from the interview with the patient and convey it back to the patient/relatives correctly	59%	65%	53%	65%
… negotiate treatment goals and communicate them to patient and/or family members	43%	66%	51%	62%
… discuss withholding of life-prolonging treatment (e.g., discuss DNR code)	35%	53%	30%	54%
… resolve conflicts or negotiate conflicts (e.g., around end-of-life care)	31%	26%	35%	44%
… discuss the patient’s impending death with him/her and/or family members/caregivers	29%	45%	39%	51%
… report the death of a patient to family members/caregivers	29%	45%	44%	50%
… cope with a palliative/dying patient	23%	61%	24%	62%
… discuss ACP (e.g., appointing a representative, providing information on PC)	22%	71%	14%	60%

Based on the PEAT communication domain; T1 = survey prior to the ACP class; T2 = survey following the ACP class and paper assignment

Finally, having gauged the students’ views on potential implications of the COVID-19 pandemic on ACP consultations, we first divided the responses into five main qualitative categories (see Table 5). Prior to the course (T1) opinions were clearly divided, with 31% feeling the COVID-19 pandemic could facilitate and 39% impede end-of-life conversations, while 21% had no views and a negligible 1% felt the COVID-19 pandemic would not affect the consultations. A small number of students (8%) described both expediting and limiting factors. After the course (T2), the opinions were more nuanced. Most (59%) now thought the COVID-19 pandemic would promote ACP as a topic for discussion, while a far smaller number (14%) reckoned it would hamper such conversations. Compared to T1, more respondents (14%) now described both pros and cons.

**Table 5:** Students’ answers on to the question: What is the effect of the COVID-19 pandemic on conversations pertaining to advance care planning?

The COVID-19 pandemic makes a conversation about advance care planning (ACP) …	T1 (***n*** = 138)	T2 (***n*** = 116)
Easier	31%	59%
More difficult	39%	14%
Both easier and more difficult	8%	14%
Makes no difference	1%	3%
I don’t know	21%	10%

The question was only posed to students in the 2020 cohort (online training); T1 = survey prior to the ACP class; T2 = survey following the ACP class and paper assignment

As to our qualitative evaluation, in Table 6 the five main categories we derived from the respondents’ accounts are presented. Category 1 and 2 are each subdivided into five and six subcategories, respectively. Category 1 involves factors that were thought to contribute to engaging in end-of-life-conversations. The respondents most frequently cited that confrontations with the COVID-19 pandemic, as reported on in the (social) media and through having people in their immediate surroundings fall ill, causes (palliative) patients to reflect more frequently on their own wishes regarding extended therapy. The second most frequently mentioned perception was that clinicians could utilize the COVID-19 pandemic as a starting point for end-of-life conversations. Many of the potentially hindering factors that were assigned to Category 2 mainly evolved around practical concerns: having to wear a mouth mask, having to maintain 6-ft distance during conversations, being restricted to online consultations to the detriment of face-to-face consultations, problems with scheduling ACP conversations due to the current high workload (health professionals) and care burden (informal caregivers), with delayed care due to regional COVID-19 measures also being mentioned often. Of particular note is that many respondents referred to emotional factors, such as the raised anxiety of patients, where these emotions could either prompt or stall talks about end-of-life wishes. Category 3 comprises all responses in which students were unable to come up with any concrete implications of the pandemic and the responses of students who misinterpreted the question such as,. Category 4 is all about logistical challenges associated with ACP, such as arranging home nursing, home care or admissions to a palliative unit, while Category 5 covers the conviction that classes or papers are not useful since one has to learn from real-life situations in clinical practice.

**Table 6:** Qualitative codes extracted from the students’ responses

Category 1: COVID-19 pandemic acts as a facilitator	
A. Raises awareness; confrontations with the disease in the immediate environment or news/the (social) media prompts reflection	
B. Serves as a starting point for ACP discussion (for both doctor and patient)	
C. Gives doctors more opportunity to gain experience with end-of-life conversations	
D. Fear/emotions may serve as a stimulus for engaging in ACP conversations	
E. Raises attention for psychosocial well-being	
Category 2: COVID-19 pandemic act as a barrier	
A. Hampers verbal and non-verbal communication (mouth masks, social distancing, online consultations)	
B. Limits contacts with doctors	
C. Prevents family/caregivers from co-attending visits	
D. Time constraints due to workload (doctor)/care burden (caregiver) leave no room for ACP/end-of-life consultations	
E. Fear/emotions may serve as a barrier (for ACP conversations and for visits to a doctor)	
F. Causes polarization in the population: creates division and distrust among groups	
Category 3: No idea/No difference/Did not understand the question	
Category 4: Logistical challenges associated with ACP	
Category 5: Attitude towards ACP training	

## Discussion

With this study, we demonstrate that the current PC/ACP training program is able to improve our students’ self-reported confidence regarding communication skills. The MD students participating in this study are only one step away from their internships and immediate contact with (palliative) patients. In 2011, Gibbins and colleagues already stated that junior doctors are not adequately prepared for the care of dying patients [19]. Our students expressed a need for more dedicated attention to PC and ACP, which is comparable to findings obtained in Germany and the United States [16, 17]. Other studies show that the interest of medical students in acquiring PC competencies is high [20]. At the University of Antwerp, an elective course on PC is offered to the second master’s year MD students, while PC is also briefly addressed during rotations in, for instance oncology, pneumology and neurology. Moreover, all students at our medical school participate in thirty 3-hours mandatory experiential communication skills sessions that address topics such as active listening, information giving, breaking bad news, active listening, shared decision making, conflict management and dealing with anxious or depressed patients. One of those sessions deals specifically with ACP. The exact content of this course can be found in Additional file 2: Appendix 2.

### Self-reported confidence

The greater majority of our students rated their confidence in general communicative skills as good (most notably their empathetic abilities, sense of respect for and knowledge of different cultures and beliefs, and informed consent issues). However, when it came to communication skills specific to PC and ACP (e.g., their ability to cope with a palliative/dying patient, discussing ACP, and negotiating treatment goals), their scores before and after the PC training course reflected far less assuredness. In their 2019 study among medical undergraduates in the Netherlands, Pieters et al. report similar findings, most notably that the current curriculum at the time paid too little attention to PC, where the students showed a particular lack of confidence in integrating the spiritual aspect of PC [21]. Effective communication training has been shown to improve students’ knowledge, attitudes, confidence, empathy, patient-centeredness, interview structure and patient satisfaction [22]. We indeed found that after having completed the PC/ACP classes, our respondents felt more competent in initiating a conversation about ACP than before the training course. However, some topics remained difficult, indicating that these specific skills warrant more attention and training during the internships, especially since at this stage of their careers students may underestimate the power of good communication skills in clinical practice. Even when clinical expertise is still developing, verbal and interactive competency can help overcome insecurities and foster a mutually beneficial patient-doctor relationship.

### Recommendations

In this section, we focus on the three aspects that were indicated by the students as aspects in which they felt least confident, in particular: discussing DNR-codes, discussing the patient’s impending death and resolving conflicts.

First of all, although the self-perceived confidence in discussing the option of a DNR code had improved following the training course, the numbers show there is still ample room for improvement. Clearly, the DNR code has always been a sensitive, emotionally charged subject in every hospital or residential care facility, but during the ongoing COVID-19 pandemic, it has become an even more urgent matter. The students’ trepidations around the issue were more than justified, since the timing and content of a DNR discussion poses distinct challenges. First and foremost, there is the medical aspect, where underlying co-morbidities and chance of recovery play an important role in the decision to expand, continue or restrict therapy for certain patients, which requires communicative competence to convey clearly. But when to broach the subject is another matter and the discussion is regularly postponed or not initiated at all. Although training how to communicate this message can already be helpful for students, it goes without saying that this is a competency that students will develop with time and much practice. We will be launching a new survey to explore the experience of attending physicians and physician assistants with end-of-life and DNR discussions in clinical practice to try and improve the curriculum and prepare students and interns better.

Secondly**,** the respondents also indicated to find it difficult to inform relatives of the impending or actual death of a loved one. Being another core task that can and should be practiced in a training context using simulation, we added this topic as a new scenario to the simulation class on “breaking bad news” [23].

Lastly and not unexpectedly, confidence scores in resolving or negotiating conflicts were relatively low. During the classes on diversity, we provide starting doctors with tools to help them deal with issues arising from differences in cultures, religions and norms and values, where in the simulation we confront them with an angry or aggressive patient. Despite this simulation exercise and as some of our students indicated, it requires encounters in clinical practice to further develop this skill.

As alluded to above, since besides practice, the acquisition of good communication skills takes time and clinical experience, investigations to gauge PC/ACP related competencies rather than self-perceived adequacy after internships have been launched, where we will be focusing on the aspects and themes that were rated as most challenging (most specifically the DNR code) to thus be able to anticipate on these aspects in our training programs. As Frey and co-authors concluded, to be able to effectively do so, dedicated, standardized tools need to be developed and validated [18].

### Face-to-face vs online training

Importantly, in our study face-to-face and online teaching showed similar trends in confidence scores, suggesting that internet-based communication skills training forms a good alternative for traditional practice classes. Requiring fewer teachers than face-to-face training, we, nevertheless, feel that the latter are better suited for training this essential competency, especially if we want that raised confidence levels are transferred into better communicative behavior in real practice.

### The impact of COVID-19 on ACP

The question on the implications of the COVID-19 pandemic for ACP showed that the students in our 2020 cohort initially mostly saw practical obstacles for end-of-life conversations such as time constraints, restrictions regarding face-to-face contact and family members visiting, and mouth masks affecting both verbal and non-verbal communication. After having studied a reflective article and discussing views in breakout rooms, more students reflected on the potential of the COVID-19 pandemic in fostering ACP, mentioning among other aspects, the patients’ and caregivers’ heightened self-reflection and the restriction on receiving visitors in the hospital. With this highly topical class, we sought to raise the students’ awareness of events and situations they could utilize as a starting point for entering into end-of-life conversations.

### Limitations

It needs to be noted that our study may be limited in that in our first (2019) cohort, the number of students completing the post-course survey (T2) was much lower than the number for the baseline assessment survey (T1). This could imply a selection bias, with only students with a special interest in PC responding at T2*.*

Also, the results presented reflect the state of affairs at our university and relate to our specific PC program, where data of our respondents are not necessarily generalizable to second master’s year MD students elsewhere, given that views and values concerning PC and ACP vary per country and for students from different cultures and origins. However, this is nuanced, given that the medicine curriculum within the different Flemish Universities is comparable, also the international studies mentioned above show similar results.

We also note that although many communication trainings are with simulation patients, the ACP training is not. Possibly, additional training with simulation patients around the indicated difficult topics would be more effective to increase their self-confidence. Still, it is encouraging that this also happens in sessions without simulation patients.

Finally, although (online) surveys can provide a quick method of research, it is not always straightforward to yield meaningful results. It is not clear to what extent students are unconsciously competent, and to what extent consciously competent of their communicative skills. Our study only describes a self-reported confidence and not a skill rating. Interestingly, a study by Graf and colleagues described that communication skills training had improved the students’ self-confidence. However, this increase in self-confidence didn’t correlate with the external rating (by patients) of their communicative skills, showing again the importance of skill rating [24]. As a side note, we would like to emphasize that our study focuses purely on the communicative aspect of ACP and the communicative skills involved. These lessons do not allow enough time and opportunities to address the whole palliative aspect and all domains of palliative care. As stated earlier, palliative care is a much broader aspect than just communicative skills alone.

## Conclusion

Our survey revealed that second master’s year MD students lack confidence in their abilities to discuss some of the most essential but sensitive topics with palliative patients and their partners/caregivers and relatives. Despite a targeted communication skills training course, end-of-life conversations remained challenging. Based on the results, the program was adapted to help improve the students’ skills in this area, where it is recommended to continue theme-based practice classes during rotations and internships, with a special focus on initiating and conducting end-of-life and DNR conversations. Although the results for the face-to-face and online training classes were comparable, classroom and practice-based training remain important.

## Supplementary Information

**Additional file 1: Appendix 1.** Part 1: Demographics. Part 2: Knowledge questions. Part 3: PEAT 7 – Communication. Part 4: COVID-19 pandemic influences ACP.

**Additional file 2: Appendix 2.** Curriculum and ACP training class.

## Data Availability

The datasets used and analyzed in the study presented are available from the corresponding author on reasonable request.

## Abbreviations

ACP: Advance care planning
AND: Allow natural death
COVID: Coronavirus disease
DNR: Do not resuscitate
EAPC: European Association of Palliative Care
PC: Palliative care
PEAT: Palliative education assessment tool
