# Supplementary material for: Communication skills training in advance care planning: a survey among medical students at the University of Antwerp
Source: BMC Palliat Care. 2022 Aug 31;21:154. doi: 10.1186/s12904-022-01042-y (PMC9428387; doi:10.1186/s12904-022-01042-y)
Supplement: Supplementary file 1 — Additional file 1: Appendix 1. Part 1: Demographics. Part 2: Knowledge questions. Part 3: PEAT 7 – Communication. Part 4: COVID-19 pandemic influences ACP. [file 12904_2022_1042_MOESM1_ESM.docx]

**Appendix 1**

## Part 1: Demographics

| Gender | ☐ Male  ☐ Female  ☐ Other identification  ☐ I do not wish to answer this |
| --- | --- |
| Age | _ _ years |
| To what religious movement do you count yourself | ☐ None  ☐ Christianity  ☐ Islam  ☐ Buddhism  ☐ Hinduism  ☐ Judaism  ☐ Other: specify _____________________ |
| Specialization preference | ☐ Family medicine  ☐ Specialised medicine  Specify: _________________________ |
| Do you feel that **enough time** is spent on end-of-life related lessons (advance care planning, palliative care, palliative sedation, living wills, euthanasia,...)? ☐ Yes ☐ No | |
| Do you feel that you have gained **sufficient knowledge** during the lessons related to the end of life (advance care planning, palliative care, palliative sedation, living will, euthanasia,...)?  ☐ Yes ☐ No | |
| Do you find that you yourself are able to **choose the right time** in a care process and **independently** have a conversation about advance care planning with a patient? ☐ Yes ☐ No | |
| What did you find useful about the module around advance care planning (assignments and theory around communication lesson of December 6)   \| Knowledge video \| ☐ Useful \| ☐ Not useful \| \| --- \| --- \| --- \| \| Lesson communication \| ☐ Useful \| ☐ Not useful \| \| Assignment \| ☐ Useful \| ☐ Not useful \| | |

## Part 2: Knowledge questions

1. In the case of a code DNR3: (1 answer is correct)
   1. The patient must be resuscitated
   2. The patient is no longer resuscitated, but is transferred to intensive care if necessary.
   3. Life-terminating therapy is started.
   4. No more active treatment is given, except for comfort care.
2. A man with metastatic colon cancer is brought in to the emergency room in overall poor condition. To ensure the patient's comfort, the patient is sedated. Several hours later, the partner walks into the room with the patient's living will. This states that patient desires euthanasia. Can this be performed now?
   1. Yes
   2. No
3. Circle the correct statements: (more than 1 answer is possible)
   1. Euthanasia can only be requested if you have less than 3 months to live.
   2. A patient with Parkinson’s disease requests euthanasia because of psychological suffering. In order for this request to proceed correctly, not only a second, but also a third independent physician should be consulted.
   3. If the patient can no longer write the request for euthanasia, the partner may do so.
   4. Euthanasia is, by law, a natural death.
4. A doctor… (1 answer is correct)
   1. … can refuse to perform euthanasia.
   2. … may perform euthanasia without consulting a second physician if the case is clear.
   3. … may perform euthanasia on patients in reversible coma who have made a previous living will.
   4. … can only perform euthanasia if a living will has been prepared.
5. Circle the correct statements: (more than 1 answer is possible)
   1. To be entitled to a palliative premium, you do not have to have a palliative status; a poor prognosis is sufficient.
   2. Home counseling with the help of a palliative care team is fully reimbursed.
   3. If you have a palliative status you no longer have to pay a remittance.
   4. Palliative care leave can only be obtained if you are the patient's partner.

## Part 3: PEAT 7 – Communication

The current curriculum covers various communication skills that are useful when communicating with patients in a palliative setting. Which ones do you think are sufficiently covered (i.e., which skills do you think you have sufficiently)?

| **I feel confident that I am able to…** | **Agree** | **Disagree** |
| --- | --- | --- |
| … cope with a palliative/dying patient | ☐ | ☐ |
| … respect and convey knowledge about different cultures and beliefs | ☐ | ☐ |
| … empathically engage in an interview/consultation | ☐ | ☐ |
| … derive relevant clinical information from the interview with the patient and convey it back to the patient/relatives correctly | ☐ | ☐ |
| … empathize with the patient and/or family members/caregiver(s) | ☐ | ☐ |
| … cooperate and communicate well within a multidisciplinary team | ☐ | ☐ |
| … negotiate treatment goals and communicate them to patient and/or family members | ☐ | ☐ |
| … resolve conflicts or negotiate conflicts (e.g., around end-of-life care) | ☐ | ☐ |
| … break bad news/conduct a ‘bad news’ consultation | ☐ | ☐ |
| … ask for informed consent | ☐ | ☐ |
| … discuss withholding of life-prolonging treatment (e.g., discuss DNR code) | ☐ | ☐ |
| … discuss ACP (e.g., appointing a representative, providing information on PC) | ☐ | ☐ |
| … discuss the patient’s impending death with him/her and/or family members/caregivers | ☐ | ☐ |
| … report the death of a patient to family members/caregivers | ☐ | ☐ |

## Part 4: COVID-19 pandemic influences ACP…

The COVID-19 pandemic makes a conversation about advance care planning (ACP)
☐ Easier
☐ More difficult
☐ Both easier and more difficult
☐ Makes no difference
☐ I don’t know
 **Explain:**
