# Supplementary material for: Communication skills training in advance care planning: a survey among medical students at the University of Antwerp
Source: BMC Palliat Care. 2022 Aug 31;21:154. doi: 10.1186/s12904-022-01042-y (PMC9428387; doi:10.1186/s12904-022-01042-y)
Supplement: Supplementary file 2 — Additional file 2: Appendix 2. Curriculum and ACP training class. [file 12904_2022_1042_MOESM2_ESM.docx]

**Appendix 2 – Curriculum and ACP training class**

| **Medicine curriculum at the University of Antwerp** | |
| --- | --- |
| Bachelor  Year 1-3 | Basic anatomy, physiology and normal organ function.  + each year six 3-hour training communication skills classes |
| Master Year 1-3 | Specific organ pathologies and application of knowledge in clinical practice.  + each year six 3-hour training communication skills classes |

In Belgium, the 6-year medicine program consists of a 3-year bachelor’s degree program, where the focus lies on normal organ function and physiology, and a 3-year master’s program that focuses on different pathologies and the application of knowledge in clinical practice. To provide students starting their practical training at the University of Antwerp with tools to develop adequate communication skills, each academic year comprises communication skills practice implemented as six 3-hour training classes following the CanMEDS "communicator" competency scenario.

| **ACP training class** |
| --- |
| Part 1: Plenary session with knowledge video  Belgian legislation on PC, ACP and euthanasia  How to define a patient as ‘palliative’  Content and implications of a declaration of intent  Part 2: Paper assignment  Reflection on own wishes and standards regarding ACP  Identifying tipping points to start a conversation about ACP  Part 3: Experiental communication skills session  Practice groups including approximately 15 students |

The ACP training class is one of the communication skills classes in the 2^nd^ master year and uses a "flipped classroom" approach. In a plenary session the students watch a knowledge video expounding among other issues, how to define a patient as ‘palliative’ (surprise question in paper assignment, see below), the content and implications of a declaration of intent, palliative sedation and euthanasia, while paying explicit attention to Belgian legislation on PC, ACP and most specifically euthanasia. In the subsequent paper assignment, we ask the students to reflect on their own wishes and standards regarding ACP. Next, the students practice communicative skills discussing various aspects of ACP, such as how to deal with or introduce the topic of a do-not-resuscitate (DNR) code or an allow-natural-death (AND) order, in practice groups including approximately 15 students. Lastly but importantly since the timing of this issue remains challenging, students learn to identify the tipping points that make it easier for doctors to start a conversation about ACP, where initially apparent barriers can be transformed into opportunities to broach the subject.
